# DHEA Attenuates Microglial Activation via Induction of JMJD3 in Experimental Subarachnoid Haemorrhage

**DOI:** 10.1186/s12974-019-1641-y

**Published:** 2019-11-28

**Authors:** Tao Tao, Guang-Jie Liu, Xuan Shi, Yan Zhou, Yue Lu, Yong-Yue Gao, Xiang-Sheng Zhang, Han Wang, Ling-Yun Wu, Chun-Lei Chen, Zong Zhuang, Wei Li, Chun-Hua Hang

**Affiliations:** 1 Department of Neurosurgery, Nanjing Drum Tower Hospital Clinical College of Nanjing Medical University, Nanjing, 210008 Jiangsu China; 2 Department of Neurosurgery, Nanjing Drum Tower Hospital, The Affiliated Hospital of Nanjing University Medical School, Nanjing, 210008 Jiangsu China; 3 Department of Neurology, Jinling Hospital, Nanjing Medical University, Nanjing, China; 4 Department of Neurosurgery, Beijing Friendship Hospital, Capital Medical University, Beijing, 100032 China

**Keywords:** Dehydroepiandrosterone, Microglia, JMJD3, Subarachnoid haemorrhage, Inflammation

## Abstract

**Background:**

Microglia are resident immune cells in the central nervous system and central to the innate immune system. Excessive activation of microglia after subarachnoid haemorrhage (SAH) contributes greatly to early brain injury, which is responsible for poor outcomes. Dehydroepiandrosterone (DHEA), a steroid hormone enriched in the brain, has recently been found to regulate microglial activation. The purpose of this study was to address the role of DHEA in SAH.

**Methods:**

We used in vivo models of endovascular perforation and in vitro models of haemoglobin exposure to illustrate the effects of DHEA on microglia in SAH.

**Results:**

In experimental SAH mice, exogenous DHEA administration increased DHEA levels in the brain and modulated microglial activation. Ameliorated neuronal damage and improved neurological outcomes were also observed in the SAH mice pretreated with DHEA, suggesting neuronal protective effects of DHEA. In cultured microglia, DHEA elevated the mRNA and protein levels of Jumonji d3 (JMJD3, histone 3 demethylase) after haemoglobin exposure, downregulated the H3K27me3 level, and inhibited the transcription of proinflammatory genes. The devastating proinflammatory microglia-mediated effects on primary neurons were also attenuated by DHEA; however, specific inhibition of JMJD3 abolished the protective effects of DHEA. We next verified that DHEA-induced JMJD3 expression, at least in part, through the tropomyosin-related kinase A (TrkA)/Akt signalling pathway.

**Conclusions:**

DHEA has a neuroprotective effect after SAH. Moreover, DHEA increases microglial JMJD3 expression to regulate proinflammatory/anti-inflammatory microglial activation after haemoglobin exposure, thereby suppressing inflammation.

## Introduction

Subarachnoid haemorrhage (SAH) is a fatal neurovascular disease with a similar disability-adjusted life years as more common types of stroke [1]. Early brain injury (EBI), characterized by sterile inflammation and neuronal damage, is the main cause of neurological impairment in SAH. Our previous studies found that remission of inflammation in EBI could improve neurological recovery [2]. Inhibition of inflammation is a promising method to reduce EBI and improve long-term prognosis, but effective therapies are lacking.

Microglia, the resident innate immune cells in the central nervous system (CNS), play a crucial role in sterile inflammation. Upon stimulation, these cells activate and produce a variety of inflammatory factors that aggravate the inflammatory response. As inflammation progresses, microglia gradually change their functions in favour of suppressing the inflammatory response and orchestrating neuronal restorative processes [3]. Microglial activation has also been found in SAH patients [4] and animal models [5]. The paradoxical roles of microglia make the switch from a proinflammatory to antiinflammatory state a potential target for inhibition of inflammation in EBI.

Dehydroepiandrosterone (DHEA, 3β-hydroxy-5-androsten-17-one) and its conjugate ester are the most abundant steroid hormones in circulation and are even more abundant in the CNS [6]. In humans, peripheral DHEA is synthesized by the adrenals and the gonads; DHEA is able to cross the blood-brain barrier (BBB) due to its lipophilic nature [7]. Moreover, in the brain, both astrocytes and neurons can synthesize DHEA [8], making the DHEA concentration much higher in the brain (13.1~29.4 nmol/L) than in the circulation (1.83 nmol/L) [6].

DHEA has been widely used as an anti-ageing health care product and is thought to play a beneficial role in many pathological changes, including neurodegeneration [9], neuropsychiatry [10], and neuroinflammation [11]. DHEA treatment has been shown to alleviate dopamine depletion in a Parkinson’s disease (PD) mouse model [12], suggesting a neuroprotective effect of DHEA. Furthermore, DHEA reportedly inhibits activation of lipopolysaccharide (LPS)-stimulated microglia, which implies an anti-inflammatory property of DHEA [11]. By binding with many receptors, such as the N-methyl-D-aspartate (NMDA) receptor [13], G protein-coupled receptors [14], and tropomyosin-related kinase A (TrkA) receptor [15], DHEA initiates different signalling pathways to play an anti-inflammatory role. Considering that the inflammatory response is a common pathological feature of these diseases and the important role of microglia in this response, we speculate that DHEA may have a regulatory effect on microglia in EBI.

To address this hypothesis, we explored the protective effects of DHEA after SAH in vivo and discussed the specific mechanisms by which DHEA regulates microglial activation in vitro.

## Materials and methods

All animal procedures were approved by the Ethics Review Committee for Animal Experimentation at Nanjing Drum Tower Hospital and were conducted in accordance with recommendations from the National Institutes of Health’s *Guide for the Care and Use of Laboratory Animals*.

### Mice and model

A total of 91 adult male C57BL/6 mice (8–10 weeks old, 20–25 g), 5 females at 18 days of gestation, and ~ 100 pups within the first day of life from Animal Core Facility of Nanjing Medical University were used. Animals were allowed ad libitum access to food and water. Mice were kept in specific pathogen-free (SPF) and comfortable conditions (12-h light/dark cycle, temperature at 25 °C, and humidity at 65%) throughout the experiment. All efforts were made to minimize the number of animals killed and their suffering in the study. Animals were randomly divided and assigned to experimental groups, and the mortality rates were 0 (0/18) in the sham group, 18.42% (7/38) in the SAH group and 11.11% (4/36) in the SAH + DHEA group (Additional file 1: Figure S1).

The endovascular perforation procedure [16] was performed by the same skilled experimenter (T.T.) (Additional file 1: Figure S2). Mice were anaesthetized with 3% and 1.5% isoflurane inhalation (RWD Life Science, Shenzhen) for induction and maintenance. Body temperature was maintained at 37 ± 0.5 °C throughout the operation. After satisfactory anaesthesia, an ~ 1.0-cm-long median cervical incision was made. A marked 6-0 filament was inserted retrogradely from the external carotid artery into the cranium through the internal carotid artery, reaching the origin of the right middle cerebral artery. Then, the filament was progressed forward quickly to puncture the artery in the SAH group or immediately withdrawn in the sham group. All mice were given 1 ml of normal saline intraperitoneally after surgery.

The neurological score was blindly evaluated on postoperative day (POD) 1 and POD 7 using a modified Garcia score by two independent observers (GJ.L. and CL.C.). Animals scoring ≤ 6 or ≥ 15 at POD1 were excluded.

DHEA (80 mg/kg) (Yuanye Bio-Technology, Shanghai) or vehicle was injected intraperitoneally once a day [11]. The surgery was performed 1 h after the second administration, and mice were executed after the third administration except the mice in neurological test (consecutive administration until POD7). Drug was administered by Y.Z., and others were blinded to the group allocation.

### Enzyme-linked immunosorbent assay

The serum and the tissues from the cortex, piriform cortex, hippocampus and hypothalamus were collected after heart perfusion and homogenized (100 mg in 1 ml 1 × PBS, according to the manual). Cell supernatants from different treatment groups were collected and tested. ELISA (Cusabio, China) was conducted according to the manual.

### Cell culture

For neuron culture, the cortex was harvested from embryonic day 18 mice after careful removal of the leptomeninges and digested with 0.25% trypsin for 10 min in a 37 °C incubator. The digestion was stopped with 2-ml foetal bovine serum (FBS, biological industries, USA), and the medium was removed. The cortices were gently titrated in medium, and the upper single-cell medium was transferred to a new tube. The gentle titration and medium transfer were repeated 3–4 times. The resulting single-cell medium was centrifuged at 1000 rpm for 5 min, and the pellet was resuspended with Dulbecco’s Modified Eagle Medium (DMEM, Gibco, USA). Eventually, neurons were seeded onto poly-d-lysine-precoated plates at a density of 7.0 × 10^5^/ml and maintained at 37 °C and 5% CO_2_. Four hours later, the medium was completely replaced with neurobasal medium containing 2% B27 and 1% GlutMax (Gibco, USA). Neurons were usually available at 7 days in vitro (DIV7). Neuronal morphology was measured by Sholl analysis with ImageJ (*n* = 5 neurons from each condition) [17].

For primary microglia culture, the cortex was harvested from neonatal mice and digested as described above. Mixed glial cells were cultured together with DMEM containing 10% FBS and 1% penicillin-streptomycin (Gibco, USA). Microglia were usually available at DIV10 to DIV14. After the plates were gently shaken, floating microglia were transferred to new plates.

### Haemoglobin-induced in vitro SAH model

Hb in bovine erythrocytes (Sigma, USA) was dissolved in complete culture medium at a concentration of 25 μmol/L. Cells were pretreated with 10^−7^ mol/L DHEA dissolved in ethanol or ethanal alone 24 h before Hb exposure. GSK-J4 or GW 441756 was added 30 min before DHEA.

### Cell viability assay by Cell Counting Kit-8

Neurons and microglia were plated in 96-well plates at a density of 1 × 10^4^ cells per well. After proper treatments, the medium was replaced completely with normal culture medium and 10 μl CCK8 (Dojindo Laboratories, Japan). After a 1-h incubation at 37 °C, the absorbance was detected at 450 nm in a microplate reader (Tecan, Switzerland). The relative cell viability was calculated according to the manual.

Conditioned medium (CM) was collected from treated microglia and filtered through a 0.22-μm filter. DIV7 neurons were cultured with the CM for 24 h and detected by Cell Counting Kit-8 (CCK8) as described.

### Real-time PCR

Total RNA was prepared with Trizol (Invitrogen, USA) according to the manufacturer’s instructions. cDNA was reverse transcribed from mRNA with reverse transcription mix (Vazyme, Nanjing). qPCR was performed with SYBER Green mix (Roche, Switzerland) using the PCR system (Applied Biosystems, USA). The results were analysed with the 2^−∆∆Ct^ method, while *18S* RNA was used for normalization. Primers used in qPCR are listed in Additional file 1: Table S1.

### Immunofluorescence staining

Frozen sections of 10-μm thickness and cells were fixed with 4% paraformaldehyde, permeabilized with 0.3% Triton X-100, and blocked with 1% BSA. Then, the sections/cells were incubated with primary antibodies at 4 °C overnight followed by incubation with proper secondary antibodies. Pictures were acquired with a confocal laser scanning microscope (FluoView FV10i, Olympus, Japan). Immunofluorescence (IF) cell count and intensity were analysed with ImageJ software (National Institutes of Health). Antibodies used in IF are listed in Additional file 1: Table S2.

### Western blotting

Brain tissues or cultured cells were lysed with RIPA (Thermo Scientific, USA) with protease inhibitor (Roche, Switzerland) and 1% phosphatase inhibitor (Sigma, USA). A bicinchoninic acid protein assay kit (Beyotime, China) was used for protein quantification. The same mass of protein was loaded onto SDS-PAGE gels for separation and then transferred to polyvinylidene difluoride membranes (Millipore, USA). Membranes were blocked with 1% BSA for 2 h at room temperature and incubated with diluted primary antibody overnight at 4 °C. Bands were analysed using ImageJ. Antibodies used in WB are listed in Additional file 1: Table S2.

### Terminal deoxynucleotidyl transferase–mediated dUTP nick end labelling

TUNEL staining was performed on frozen brain sections with a TUNEL detection kit (Beyotime, China) according to the manufacturer’s instructions. In brief, the slides were first incubated with the primary antibody anti-NeuN (1:200, 26975-1-AP, Proteintech) overnight, followed by incubation with the corresponding secondary antibody for 1 h. One microliter of TdT enzyme was diluted 5 times and mixed with 45 μl of labelling solution for one section. Each section was incubated with the mixture for 30 min at room temperature. Sections were scanned by a confocal laser scanning microscope. The whole-brain NeuN^+^ TUNEL^+^/ NeuN^+^ proportion was analysed with ImageJ (*n* = 3 brains). NeuN^+^ TUNEL^+^/NeuN^+^ proportion = (area (NeuN^+^ cells) + area (TUNEL^+^ cells)—area (positive cells in merge figure))/Area (NeuN^+^ cells) × 100%.

### Water content

The intact brains were dissected after decapitation and divided into right and left hemispheres. Each hemisphere was weighed immediately, placed in an oven for 24 h and weighed again. Water content = ((wet weight–dry weight)/wet weight) × 100%.

### Statistical analysis

All data were expressed as the mean ± standard error of the mean (SEM). All data were first tested for normality. Statistical analysis was performed using Prism 8.02 (GraphPad Software, USA). Two-tailed Student’s *t* test was used to assess differences between two groups, and one-way ANOVA followed by Tukey’s test was used for comparisons of more than two groups. Two-way ANOVA followed Dunnett’s multiple comparisons test was used in Sholl analysis. *P* < 0.05 was considered statistically significant.

## Results

### DHEA alleviates EBI after SAH in mice

**Fig. 1:**
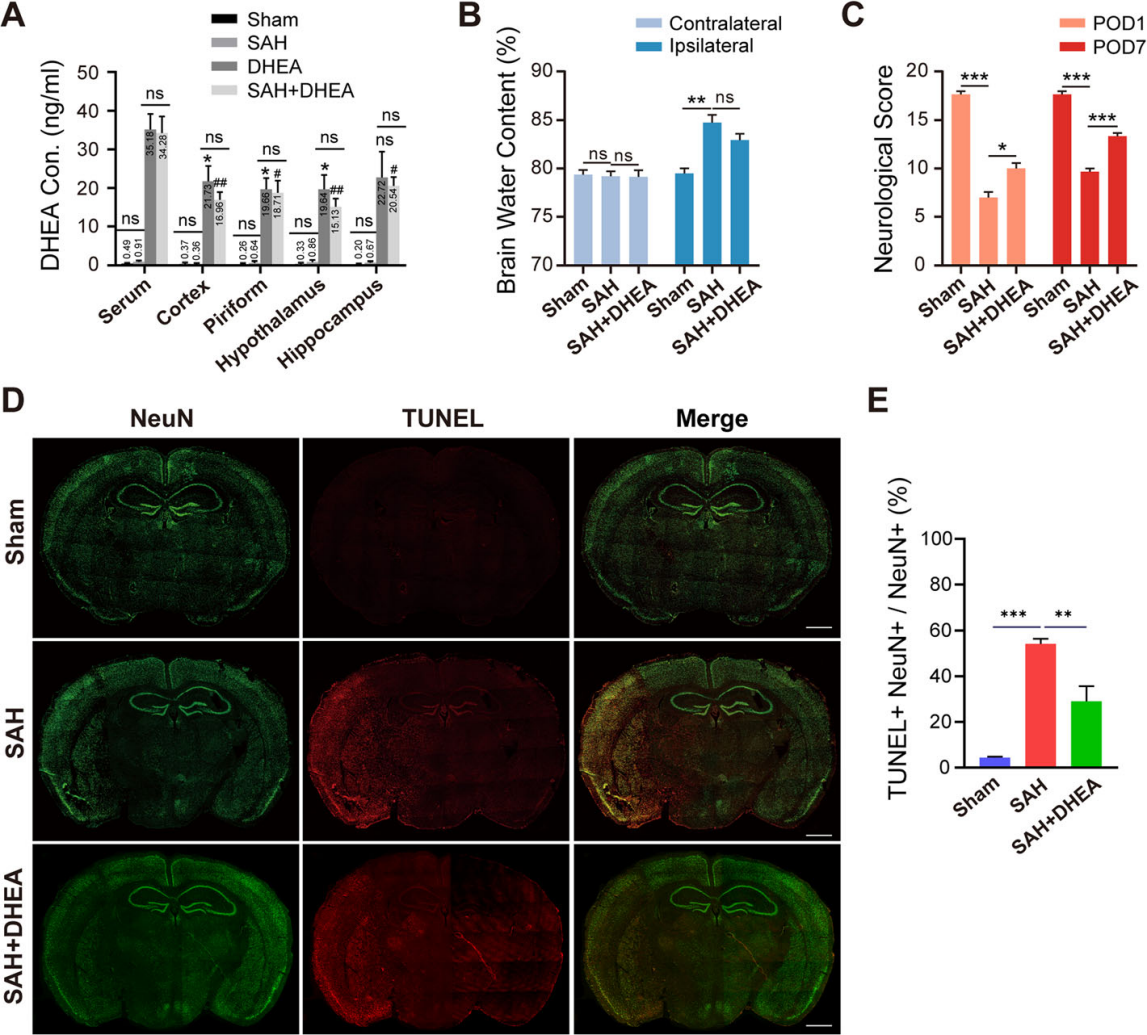
DHEA mitigated severe neuronal damage after endovascular perforation in SAH model mice. **a** After intraperitoneal injection, DHEA levels in the serum, cortex, hippocampus and hypothalamus were measured by ELISA (*n* = 5, one-way ANOVA, *P < 0.05 compared with serum in the vehicle group; #*P <* 0.05, ##*P <* 0.01 compared with serum in the SAH + DHEA group). **b, c** DHEA treatment resulted in less water content on POD 1 and better neurological test performance on both POD 1 and POD 7 (*n* = 6 in both experiments, one-way ANOVA). **d, e** Representative TUNEL images 24 h after SAH and whole-brain NeuN^+^ TUNEL^+^/ NeuN^+^ proportion (*n* = 3 brains, one-way ANOVA). POD, postoperative day; Green, NeuN; red, TUNEL; bar = 1 mm. ns, not significant, *P < 0.05, **P < 0.01, ***P < 0.001

We first tested the DHEA concentration in the serum and various parts of the brain by ELISA (*n* = 5). In the non-treated mice, the basal DHEA concentration ranged 0.20~0.49 ng/ml in the sham group and lightly higher (0.36~0.91 ng/ml) in the SAH group but no significant difference. After DHEA administration, a dramatic increase in DHEA concentration was detected up to 24 h regardless of operation (Fig. 1a). The content of DHEA in the serum (34.28–34.18 ng/ml) was significantly higher than that in the brain (15.13–22.72 ng/ml) in both the DHEA and SAH + DHEA groups, but no obvious difference was noticed among the various brain tissues. These results confirmed that peritoneal injection was a reliable route of administration and that SAH did not affect DHEA content (Fig. 1a).

As shown in Fig. 1b, d, severe parenchyma oedema and massive neuronal apoptosis were observed in the ipsilateral hemisphere after SAH (*n* = 6). Corresponding to the injury, we found an apparent compromise in the neurological test results, both at POD 1 and POD 7 (Fig. 1c, *n* = 6). These results are all indicative of severe EBI after SAH.

Mice pretreated with DHEA had remarkably lower TUNEL^+^ NeuN^+^ proportion than those in the SAH group (*n* = 3, 54.24 ± 2.24% in the SAH group versus 29.04 ± 6.66% in the SAH + DHEA group). The water content in the ipsilateral hemisphere was 80.63 ± 0.75% in the SAH + DHEA group, which was lower but not significantly than the 83.30 ± 0.81% observed in the SAH group (*n* = 6). Furthermore, DHEA improved sensorimotor functions both at POD 1 and POD 7. These results indicate that DHEA plays a neuroprotective role in SAH mice.

### DHEA inhibits proinflammatory microglial activation in vivo after SAH

**Fig. 2:**
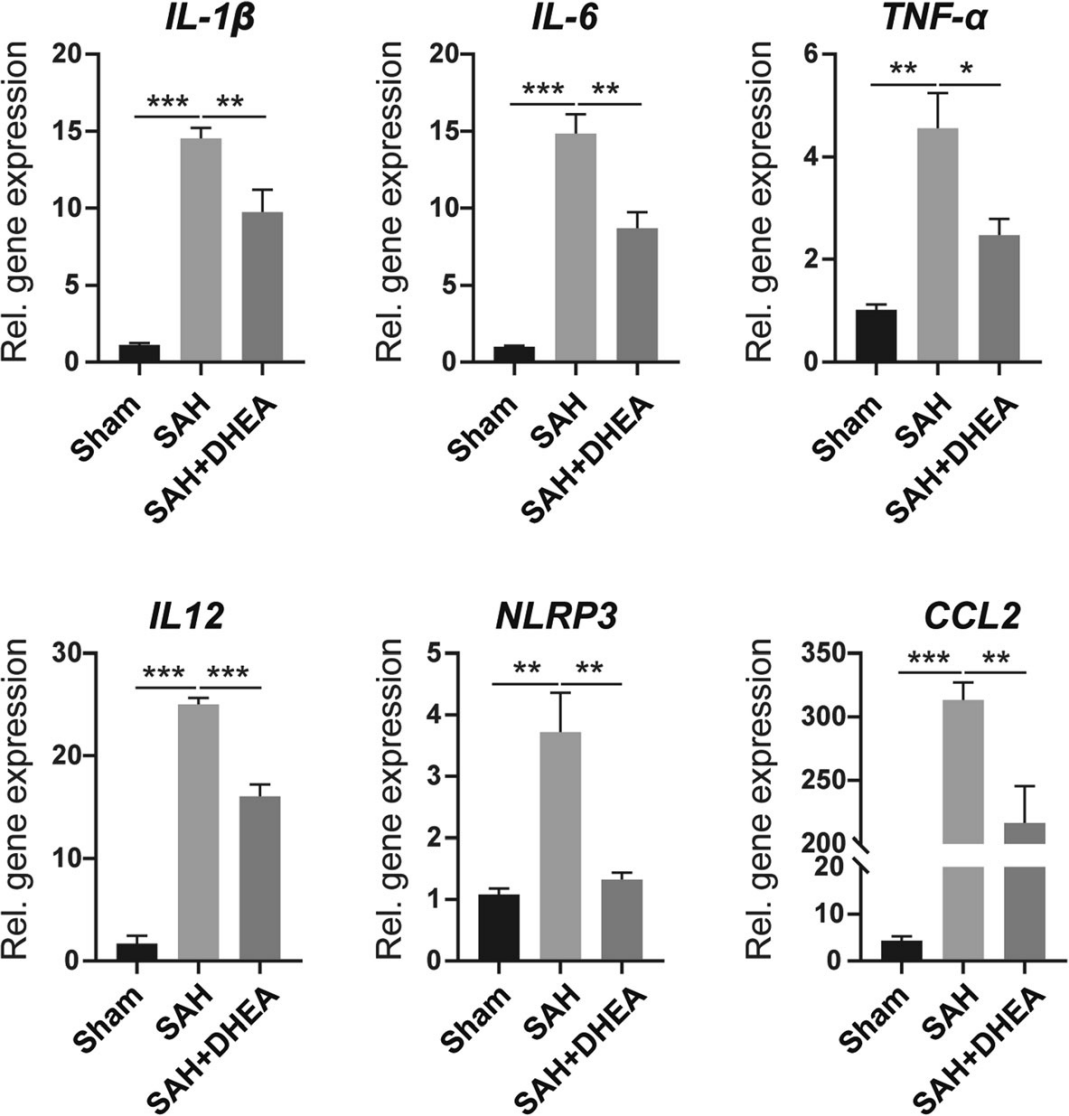
DHEA attenuated excessive inflammation after SAH in mice. The mRNA levels of *IL-1β*, *IL-6*, *TNF-α*, *IL-12*, *CCL2* and *NLRP3* from affected brain extracts were detected by qPCR (*n* = 5, one-way ANOVA, *P < 0.05, **P < 0.01, ***P < 0.001)

First, we examined inflammatory status after SAH in affected brain extracts by qPCR (*n* = 5). mRNA levels of cytokines such as *interleukin* (*IL)-1β*, *IL-6*, *tumour necrosis factor (TNF)-α*, and *IL-12*, chemokines such as C-C motif chemokine ligand 2 *(CCL2)*, and inflammasome NLR family, pyrin domain-containing 3 *(NLRP3)* were significantly higher in the SAH group than in the sham group. However, there DHEA administration remarkably attenuated these increases (Fig. 2).

To further explore the changes and role of microglia in EBI, we performed double IF staining. In the CNS, Iba-1 is a specific microglial marker independent of microglial status, whereas CD86 is a classic marker of proinflammatory activation. When microglia are activated, they transform from rod-shaped cell bodies and ramified processes to rounded amoeboid morphologies, large somas, and a short thick process.

**Fig. 3:**
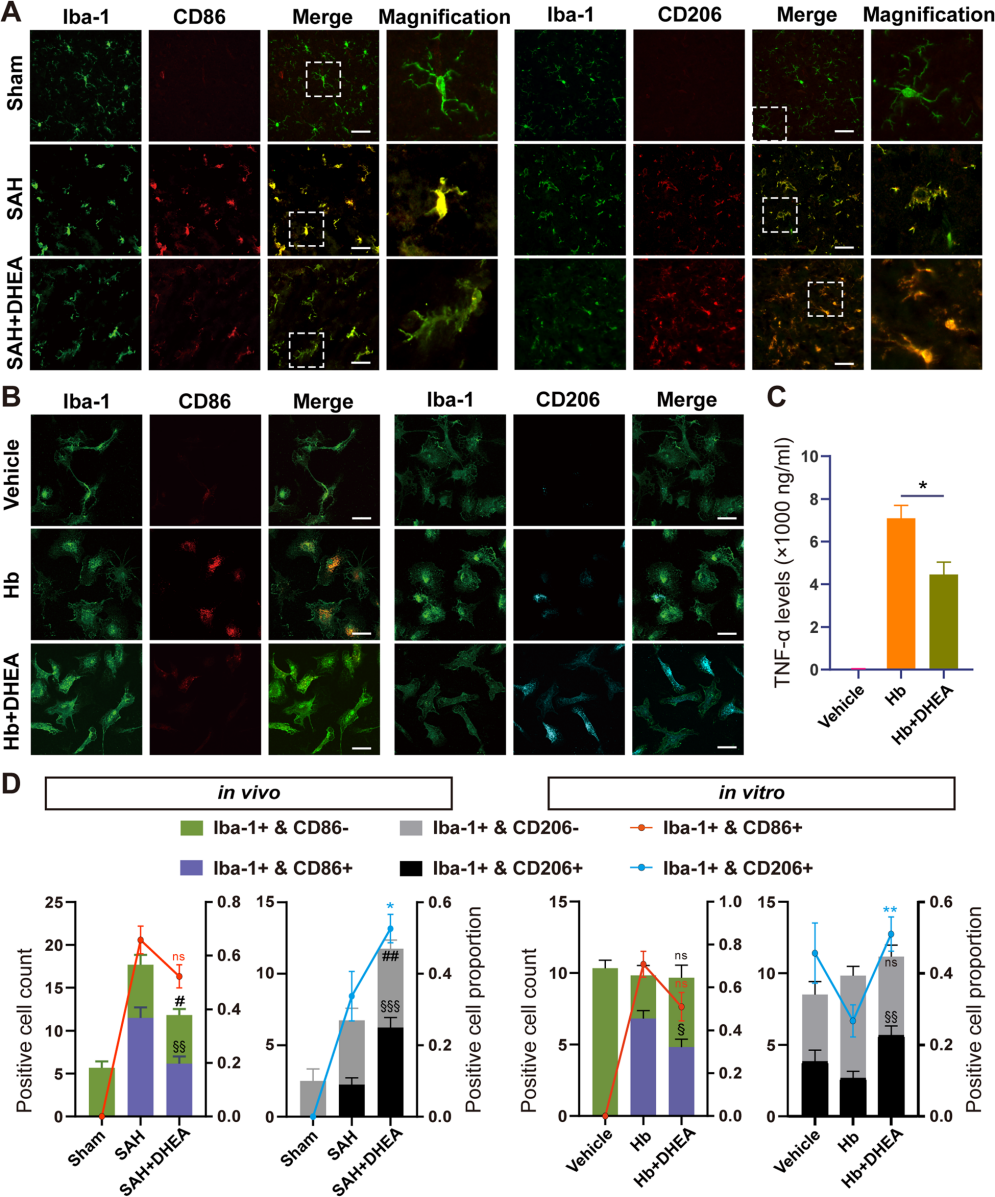
DHEA exerted an anti-inflammatory effect by regulating microglial activation in vivo and in vitro. Co-localization of Iba-1 and CD86 or CD206 demonstrated a switch from proinflammatory to anti-inflammatory activation in the presence of **a** DHEA after SAH in the piriform and **b** Hb exposure in vitro*.* Magnified views are marked with dashed line boxes. **c** TNF-α levels in the CM from various treatment microglia was tested by ELISA (*n* = 4, *t* test between Hb and Hb + DHEA). **d** The positive cell count (histogram, left *Y*-axis) and positive cell proportion (polygram, right *Y*-axis) of Iba-1^+^ and CD86^+^/CD206^+^ from **b** piriform and **c** in vitro (*n* = 6 fields, 400 × 400 μm, *t* test for comparison between SAH/Hb and SAH/Hb + DHEA; *P, #*P*, §*P* < 0.05, **P, ##*P*, §§*P* < 0.01, ***P, ###*P*, §§§*P* < 0.001, ns, not significant). Hb, haemoglobin; Green, Iba-1; red, CD86; cyan, CD206; blue, DAPI; bar = 70 μm

Significantly fewer CD86^+^/Iba-1^+^ microglia were accumulated in the affected area in the SAH + DHEA group than in the SAH group, although the proportions were not significantly different (Fig. 3a, d, *n* = 6 fields from 3 independent experiments). Moreover, a significantly higher number and percentage of CD206^+^/Iba-1^+^ microglia were detected in the SAH + DHEA group than in the SAH group (Fig. 3a, d). In addition, the microglia exhibited an intermediate morphology between rest and activation in the presence of DHEA (Fig. 3a). Together, the IF images and qPCR results indicated that DHEA may inhibit SAH-induced proinflammatory microglial activation in vivo.

### DHEA inhibits Hb-induced proinflammatory microglial activation and the immune response in vitro

Damage-associated molecular patterns (DAMPs), such as plasma proteins and Hb, stimulate microglial reactivity [18]. Primary microglia were treated with 25 μmol/L Hb with or without DHEA. After 24 h, microglia transformed into a disc shape under Hb exposure, whereas the microglia in the vehicle group remained bipolar (Fig. 3b). Hb also promoted the production and secretion of TNF-α in the medium, which suggested proinflammatory microglial activation. However, the secretion of TNF-α was restricted by DHEA (Fig. 3c, *n* = 4).

Staining for surface markers showed results consistent with those of the in vivo experiments. Compared with the Hb group, the DHEA-treated group had a significantly higher proportion of CD206^+^/Iba-1^+^ microglia but lower proportion of CD86^+^/Iba-1^+^ microglia, although the latter difference was not significant (Fig. 3b, d, *n* = 6 fields from 3 independent experiments). Taken together, these observations suggested that DHEA repressed proinflammatory microglial activation to exert an anti-inflammatory effect in vitro.

### JMJD3 is critical for the DHEA-induced mitigation of microglial inflammation after SAH

**Fig. 4:**
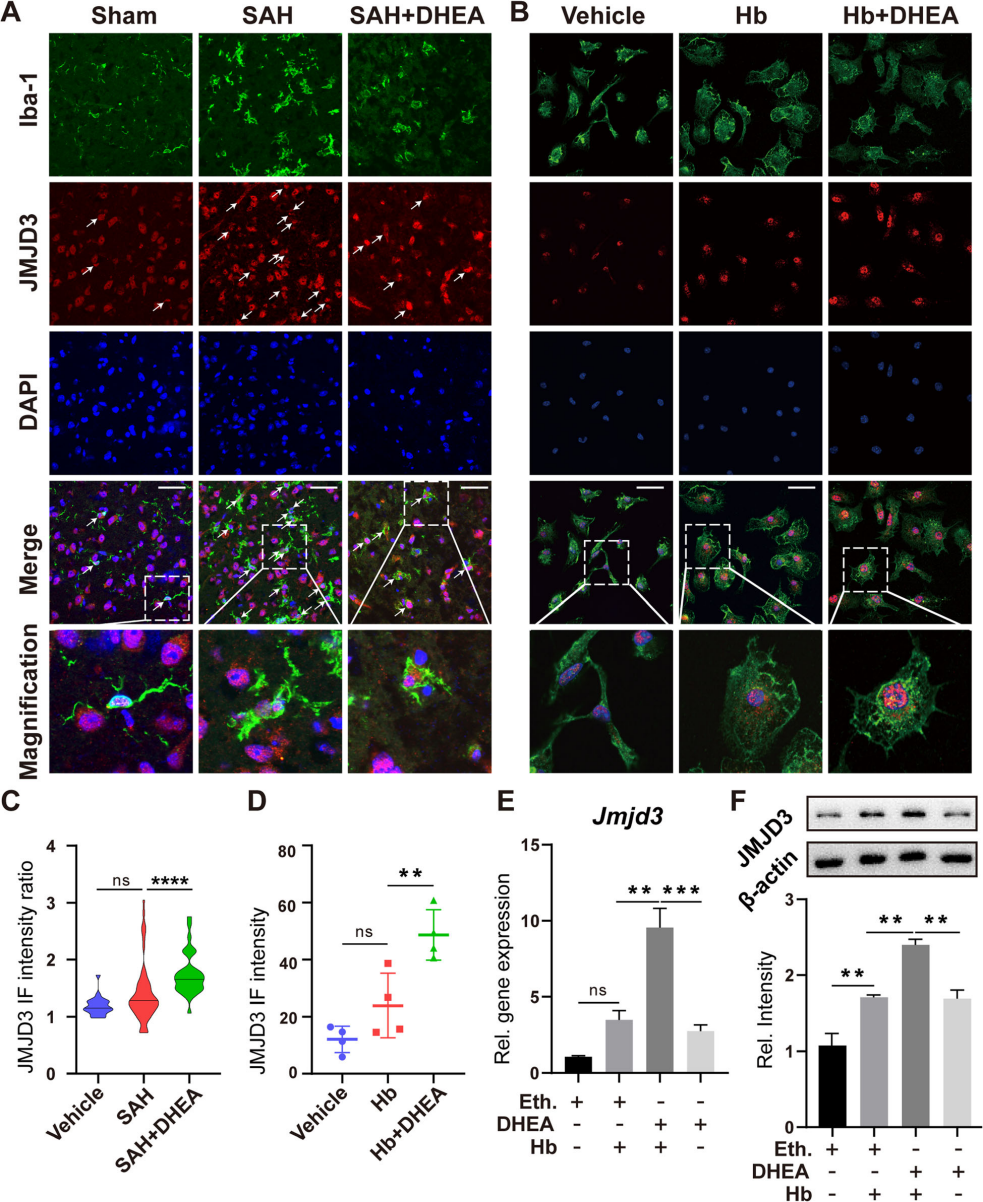
Transcription and translation of JMJD3 were induced by DHEA after Hb exposure. **a, b** Co-localization of Iba-1 and JMJD3 demonstrated an induction of JMJD3 expression after SAH/Hb exposure that was even higher in the presence of DHEA. Magnified views are marked with dashed line boxes. Green, Iba-1; red, JMJD3; blue, DAPI; bar = 70 μm. **c** The relative single cell fluorescence intensity (JMJD3 in Iba-1^+^/mean of JMJD3 in Iba-1^-^) was calculated and presented in a violin plot (*n* = 20 in sham, *n* = 79 in SAH, *n* = 43 in SAH + DHEA from 6 fields, non-specific one-way ANOVA, ****P < 0.0001). **d** Fluorescence intensity of JMJD3 after Hb exposure (*n* = 6 fields, one-way ANOVA). **e, f** qPCR and WB showed an induction of *Jmjd3* mRNA and protein expression in microglia treated with DHEA (*n* = 4 in qPCR and n = 3 in WB, one-way ANOVA), ns, not significant, *P < 0.05, **P < 0.01, ***P < 0.001. Eth., ethanol

JMJD3 is a regulator of microglial activation and may play a role in the above modulation. We first located the JMJD3 protein in microglia by IF. Colocalized with Iba-1, JMJD3 was mainly located in the nucleus, which is consistent with its function (Fig. 4a, b, *n* = 6 fields from 3 independent experiments). The JMJD3^+^/Iba-1^+^ cell count increased dramatically after SAH, but fewer double-positive cells were counted in the SAH + DHEA (Additional file 1: Figure S3). However, quantitative analysis of single-cell fluorescence showed an increase in JMJD3 fluorescence intensity within Iba-1^+^ cells after DHEA exposure, which was significantly higher than that of SAH cells (Fig. 4c). Similar results were found in vitro (Fig. 4b, d)*.*

Upon stimulation with Hb for 24 h, the mRNA and protein expression of *Jmjd3* increased significantly. After pretreatment with DHEA alone in vitro, there was also an elevation in both *Jmjd3* mRNA and protein (Fig. 4e, f). In the presence of both Hb and DHEA, a more significant increase was observed (Fig. 4e, f). Unfortunately, we did not find this elevation in the whole-brain extract (Additional file 1: Figure S4), potentially due to the small proportion of microglia in the CNS.

**Fig. 5:**
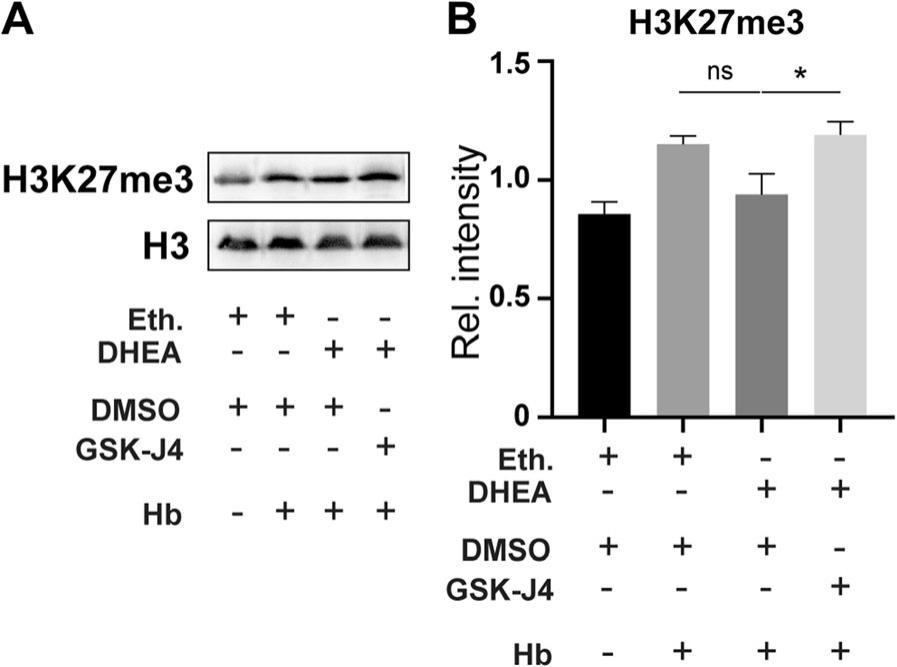
DHEA alleviated H3K27me3, and this effect was blocked with a JMJD3 inhibitor in vitro. **a, b** Methylation level at H3K27 is indicated by WB (*n* = 4, one-way ANOVA, ns, not significant, *P < 0.05)

The augmentation of JMJD3 expression after DHEA administration implies that JMJD3 might play a role in the anti-inflammatory effects of DHEA. To test this hypothesis, we examined the H3K27me3 levels with WB (*n* = 4). DHEA eliminated the H3K27me3 increase after Hb exposure, but this effect was blocked by a specific JMJD3 inhibitor, GSK-J4 (Fig. 5a, b), suggesting a critical role of JMJD3. GSK-J4 is a specific inhibitor of JMJD3 and ubiquitously transcribed tetratricopeptide repeat, X chromosome (UTX). We determined the expression of UTX after Hb and DHEA treatment and found that there was no obvious change in neurons or microglia in vitro (Additional file 1: Figure S5). Therefore, we believe that the effects of GSK-J4 on UTX can be ignored.

### Inhibition of JMJD3 in microglia abolishes the anti-inflammatory and neuroprotective effects of DHEA

**Fig. 6:**
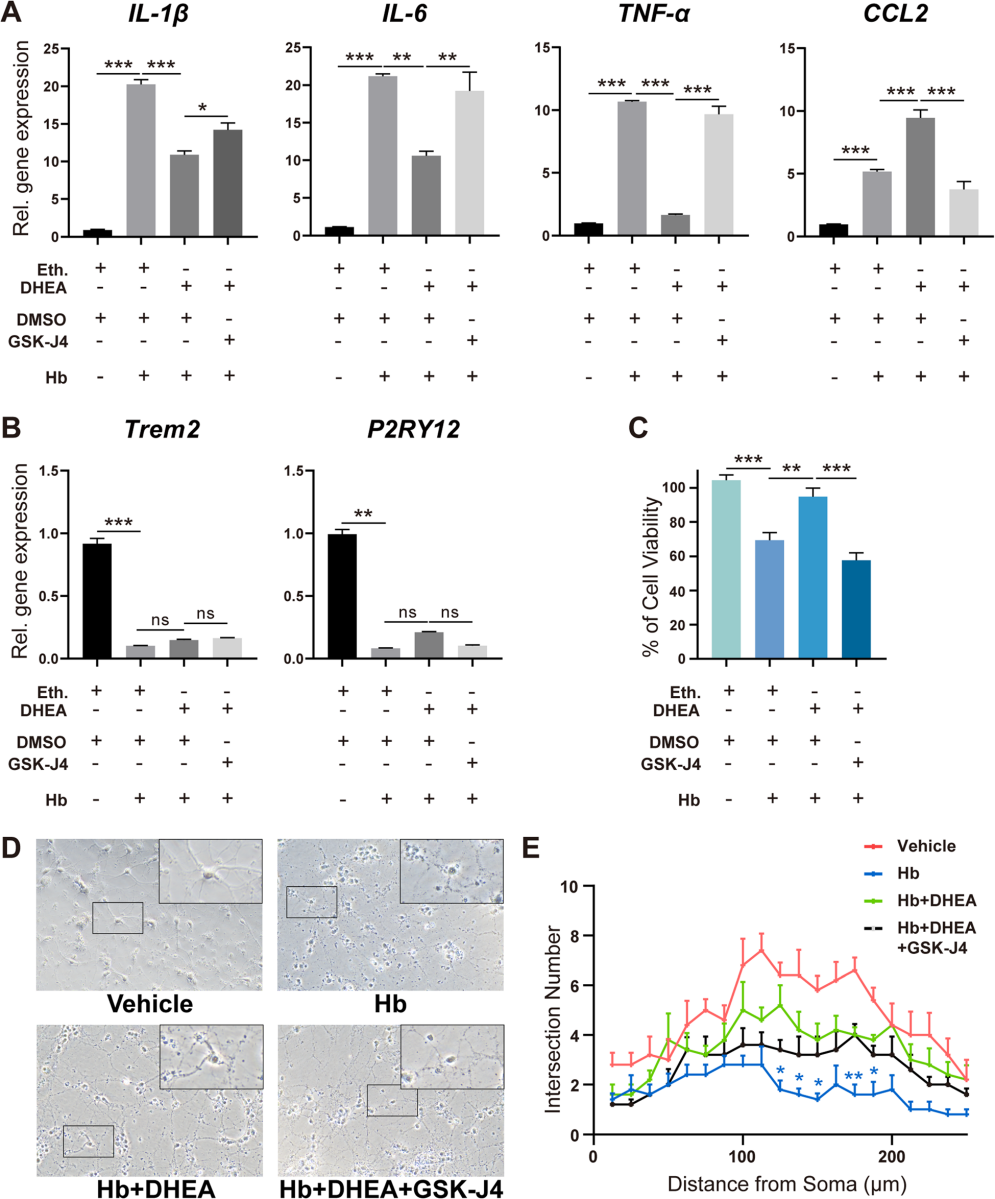
JMJD3 mediated the anti-inflammatory effect of DHEA. The mRNA expression levels of **a** *IL-1β, IL-6, TNF-α,* and *CCL2* and **b** two homeostasis genes were detected by qPCR (*n* = 5, non-parametric test for *P2RY12* and one-way ANOVA for the other). The gene expression levels in the vehicle group treated with dimethyl sulfoxide (DMSO) or ethanol were set as 1. **c** The CM collected from microglia was added to the primary neurons for another 24 h, and neuron viability was assessed by CCK8 (*n* = 5, one-way ANOVA). **d** Representative fields of primary neurons cultured with CM from microglia exposed to different treatments for 24 h. **e** Neuronal morphology was investigated with Sholl analysis (*n* = 5, two-way ANOVA with Dunnett’s multiple comparisons test, the footnote* indicates the statistically significant difference between Hb and Hb + DHEA; the difference between vehicle and Hb is not shown; no significant difference between Hb + DHEA and Hb + DHEA + GSK-J4). Bar = 50 μm. Eth., ethanol. *P < 0.05, **P < 0.01

Various inflammatory factors are important mediators of microglial sterile inflammation. We next examined the role of JMJD3 in the production of inflammatory factors. Primary microglia were treated with a proven effective concentration of GSK-J4 (10 μmol/L) 30 min before DHEA application [19]. After Hb exposure, the elevation in genes *IL-1β*, *IL-6*, and *TNF-α* and chemokine *CCL2* was validated (Fig. 6a, *n* = 5); in contrast, the expression of microglia homeostatic signatures, *Trem2* and *P2RY12* [20], was downregulated (Fig. 6b, *n* = 5). These results also demonstrated proinflammatory microglial activation after Hb stimulation. Supplementary DHEA suppressed *IL-1β*, *IL-6*, *TNF-α*, and *CCL2* expression after Hb exposure, but this suppression was blocked by GSK-J4 (Fig. 6a). Furthermore, the upregulation of *P2RY12* after DHEA was also blocked by GSK-J4, although there were no significant changes in *Trem2* expression (Fig. 6b).

In addition to the secretion of the above proinflammatory factors, microglia can also secrete several neurotrophic factors, such as brain-derived neurotrophic factor (BDNF), which act together on neurons. To explore this complex influence, we cultured primary neurons with CM from microglia exposed to different treatments (*n* = 5). The CM from Hb-exposed microglia induced ~ 30% neuron death (Fig. 6c). Importantly, neurons cultured with CM from DHEA-pretreated microglia exhibited a significantly higher cell viability of ~ 95%. This protective effect of DHEA was abolished by inhibition of JMJD3 (~ 57%) (Fig. 6c). Finally, significant improvement was also found in the DHEA group compared with that in the Hb group in the Sholl analysis, although no significant difference was found after JMJD3 inhibition (Fig. 6d, e, *n* = 6).

### DHEA promotes anti-inflammatory microglial activation after Hb stimulation

**Fig. 7:**
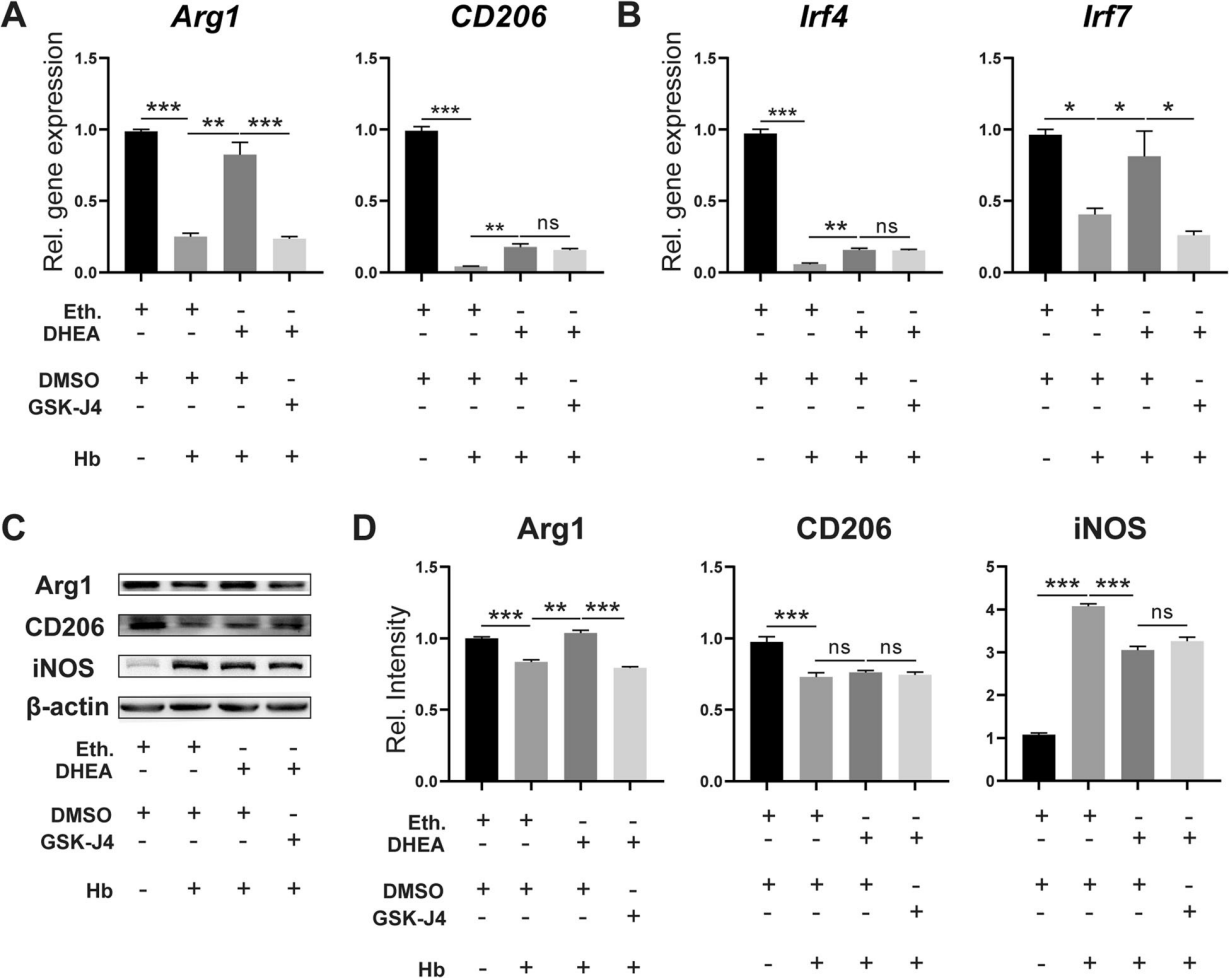
DHEA promoted microglial anti-inflammatory activation after Hb exposure. mRNA expression of **a** *Arg1, CD206* and **b** *Irf4* and *Irf7* were analysed by qPCR (*n* = 5, one-way ANOVA). **c, d** The protein levels were analysed by WB (*n* = 4, one-way ANOVA). Eth., ethanol; DMSO, dimethyl sulfoxide. ns, not significant, *P < 0.05, **P < 0.01, ***P < 0.001

Some classical anti-inflammatory markers were also detected by both qPCR and WB. Agr1 is the direct target of JMJD3 and a classical anti-inflammatory marker [21]. In cultured microglia, *Arg1* was downregulated at both the transcriptional and translational levels after Hb exposure and increased in the presence of DHEA, but the increase was blocked by GSK-J4 (Fig. 7a, c and d, *n* = 5 in qPCR, *n* = 4 in WB). DHEA also restored the mRNA level of *CD206* but failed to have a significant effect at the protein level. Inducible nitric oxide synthase (iNOS) is upregulated after stimulation and produces nitric oxide (NO) as a defence mechanism. WB showed an augmentation of iNOS protein expression after Hb exposure in microglia, but this increase was mitigated by DHEA treatment. There was a weak upregulation after JMJD3 inhibition, but this effect was not significant (Fig. 7c and d).

Interferon regulatory factor 4 (Irf4) and Irf7 are targets of JMJD3 and play a role in the regulation of macrophage and microglia activation [3, 21–23]. DHEA increased the mRNA expression of *Irf4* and *Irf7* after Hb exposure, and upregulation of *Irf7* was also inhibited by GSK-J4 (Fig. 7b, *n* = 5). These results suggested that DHEA promotes the anti-inflammatory activation of microglia and that JMJD3 may play an important role in this process.

### The TrkA/Akt pathway is involved in the induction of JMJD3 by DHEA

**Fig. 8:**
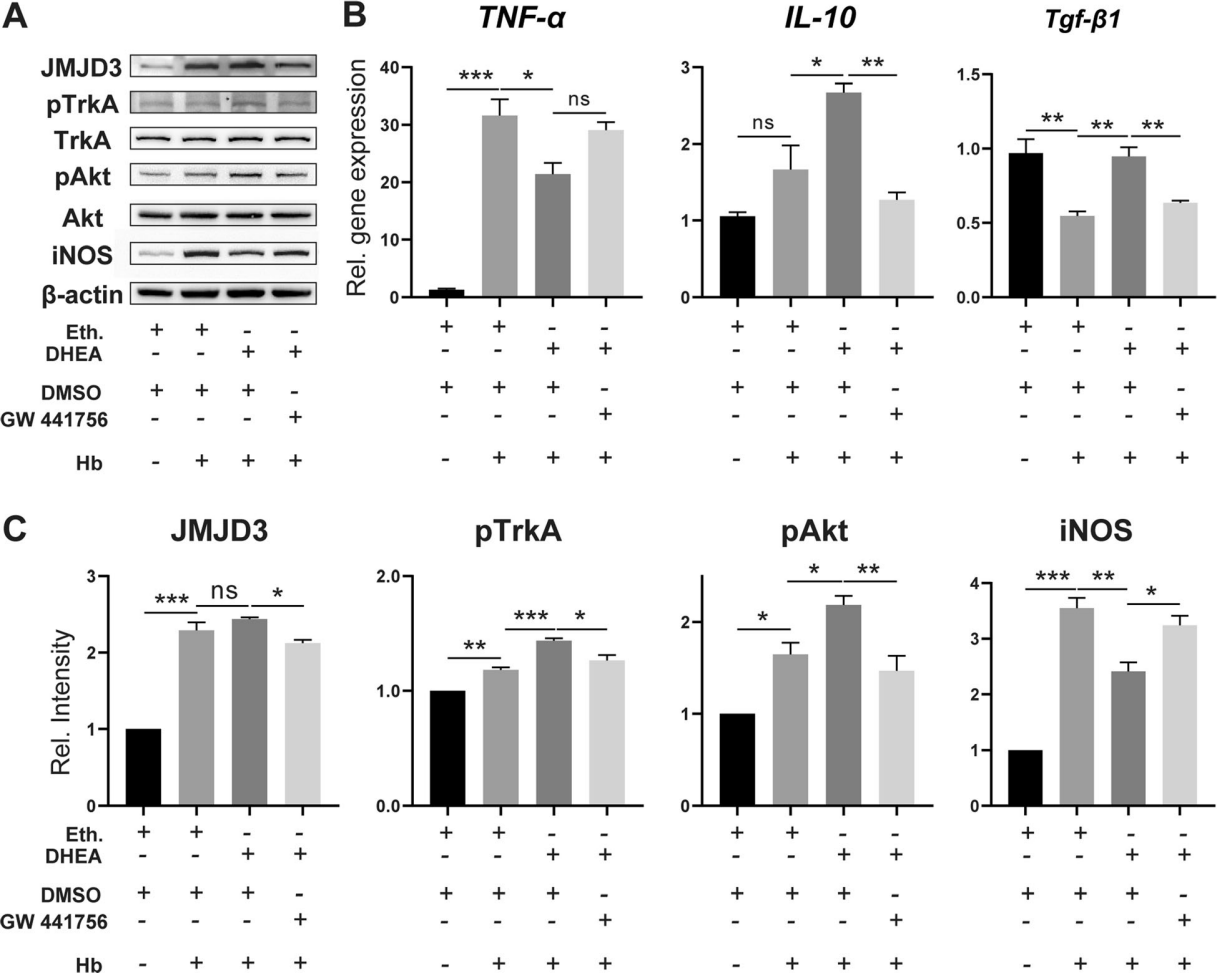
The TrkA/Akt pathway was involved in the anti-inflammatory effects of DHEA in microglia. **a**, **c** JMJD3, TrkA/phosphorylated TrKA (pTrkA), Akt/pAkt and iNOS protein expression were analysed by WB (*n* = 3, one-way ANOVA). **b** mRNA levels of *TNF-α*, *IL-10* and *Tgf-β1* were analysed by qPCR (*n* = 3, one-way ANOVA). Eth., ethanol, DMSO, dimethyl sulfoxide. ns, not significant, *P < 0.05, **P < 0.01, ***P < 0.001

DHEA is a ligand of TrkA and sufficient to initiate the TrkA/Akt signalling pathway [11, 15, 24]. Therefore, we investigated whether TrkA is also involved in the regulation of Hb-activated microglia by DHEA. WB demonstrated increased phosphorylation of TrkA and Akt after Hb treatment, which further increased after DHEA pretreatment (Fig. 8a, *n* = 3). Additionally, the protein level of JMJD3 was correlated with the phosphorylation level of TrkA and Akt. However, these changes were blocked by the TrkA inhibitor GW 441756 (Fig. 8a, c).

qPCR was further used to examine inflammatory gene expression in response to the TrkA inhibitor. The protective effects of DHEA in increasing the expression of *IL-10* and *transforming growth factor beta 1 (Tgf-β1)* were reversed by the administration of the inhibitor; however, there was no significant increase in *TNF-α* expression with the TrkA inhibitor (Fig. 8b, *n* = 3).

## Discussion

**Fig. 9:**
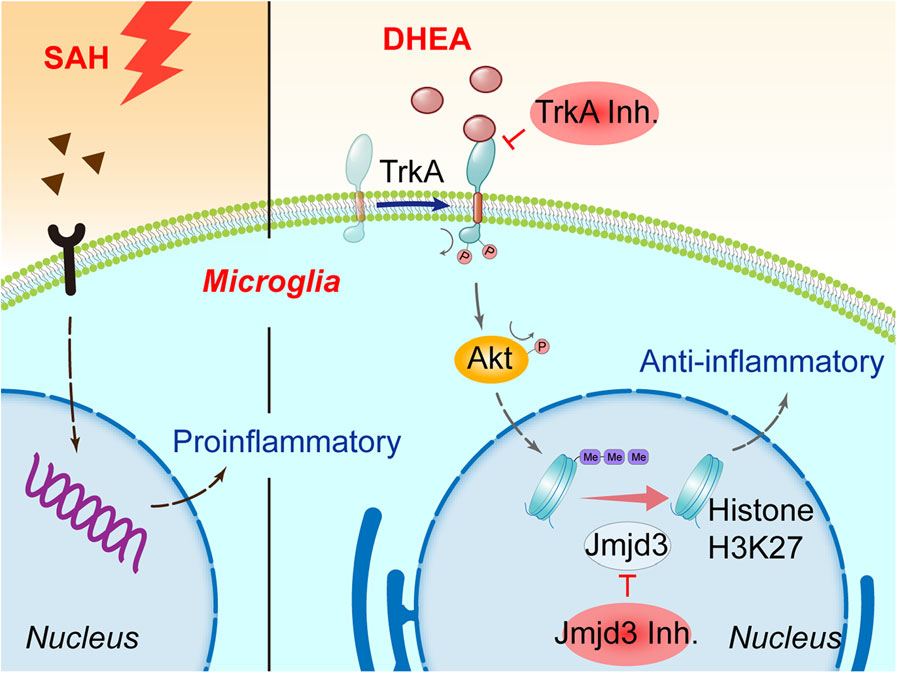
As innate immune cells in the CNS, microglia are activated after SAH in vivo and in vitro. Microglia then initiate an inflammatory cascade to aggravate neuronal damage, which is an important cause of EBI. A neurosteroid, DHEA, exerts an anti-inflammatory effect after Hb exposure by initiating the TrkA/Akt signalling pathway and inducing the expression of the H3K27me3 demethylase, Jmjd3. Jmjd3 then negatively regulates proinflammatory genes and modulates the proinflammatory/anti-inflammatory switch, possibly through its downstream transcription factors

In this study, the anti-inflammatory effects of DHEA in EBI were verified in an endovascular perforation mouse model. The major findings were as follows. First, systemic administration of DHEA increased DHEA levels in the brain and attenuated EBI after SAH. Second, DHEA administration altered microglial activation status in the ipsilateral hemisphere. Third, the expression of a H3K27 demethylase, JMJD3, was upregulated after DHEA treatment and modulated its anti-inflammatory effects. Finally, DHEA induced *Jmjd3* gene expression through the TrkA/Akt signalling pathway in vitro (Fig. 9).

More than 40% of SAH survivors experience long-term neurological impairment [25], mainly due to EBI [26, 27]. The inflammatory response is an important process in EBI. Released neuronal necrotic products, Hb, and other degradation products stimulate microglia immediately after SAH. The proinflammatory microglia initiate and amplify the inflammatory response in the acute phase, releasing a variety of inflammatory factors that further damage neurons. Our study found typical proinflammatory microglia in the neuronal damaged region but no other regions. The mRNA levels of a variety of cytokines were also elevated in the affected regions. The co-occurrence of massive neuronal apoptosis and proinflammatory microglial activation validates the important role of the microglia-triggered inflammatory responses in EBI, but neuronal damage may be both a cause and a consequence of microglial activation. Proinflammatory microglial activation aggravates neuronal damage both in ischaemic stroke [28] and intraparenchymal haemorrhage [29], whereas modulation of microglial activation has a beneficial effect. Our study revealed a similar result in SAH.

Dehydroepiandrosterone sulfate (DHEAS) is often discussed with DHEA because it is the sulfate form and a reservoir of DHEA [30]. Many studies have suggested a protective profile of DHEA/DHEAS, but the detailed mechanism is not clear. DHEAS levels have been shown to be significantly lower in Alzheimer’s disease (AD) patients than in controls [31]. In addition, DHEA blocked microglial nitrite production under a co-stimulation of amyloid β-peptide and interferon-γ in vitro [32], suggesting a potential protective effect in AD. An observational clinical study found that high serum DHEAS was associated with a decreased IL-6 serum level and a more favourable long-term neurological outcome after aneurysmal SAH [33]. This is an interesting phenomenon, but whether exogenous DHEA supplementation plays a role is unknown. Although only serum DHEA/DHEAS levels were tested in these studies, we have reasons to believe that the DHEA levels in the brain correspond with those in the periphery. These observations indicate that DHEA/DHEAS may play a protective role in the regulation of immune cell inflammatory responses, but the details are unclear.

Our experiments revealed that supplementary DHEA inhibited the proinflammatory response of microglia in SAH, decreased the expression of inflammatory factors and reduced neuronal damage both in vivo and in vitro. These results are generally consistent with previous reports. Regrettably, we did not perform a long-term observation and are unable to determine how DHEA affects microglia in the delayed phase of SAH to help brain repair and regeneration, but this will be the focus of future studies.

JMJD3 is an inducible histone demethylase [34] that promotes anti-inflammatory activation in macrophage/microglia and suppresses inflammatory responses [23]. JMJD3 in macrophages regulates the expression of molecules involved in anti-inflammatory activation by altering histone methylation levels of the transcription factor Irf4 [21], which decreased significantly after Hb stimulation and increased after DHEA treatment in our study (Fig. 7b). The demethylase also plays a role in promoting anti-inflammatory activation in microglia. Tang’s study found that inhibition of JMJD3 blocked anti-inflammatory activation and enhanced proinflammatory activation in N9 microglia and was associated with inflammatory responses in PD [22]. Together with our experimental results, these observations reveal that the JMJD3-Irf4 axis also plays a regulatory role in microglia after Hb exposure and is enhanced by DHEA treatment. Our study showed a corresponding change in *Irf4,* but further validations are needed.

TrkA is phosphorylated upon ligand binding, and TrkA phosphorylation has been shown to occur as early as minutes after DHEA administration [11], indicative of direct binding. Evidence of direct binding was also found by visualization of bovine serum albumin (BSA)-bound DHEA fluorescence [15]. In our study, the specific antagonist of TrkA, GW 441786, successfully inhibited the phosphorylation of TrkA and the subsequent increase in JMJD3 in vitro, validating the interaction between DHEA and TrkA.

There are few limitations in our study. First, the critical role of JMJD3 in the microglial modulation effect of DHEA was only validated in vitro*.* The lack of a reliable route of administration for GSK-J4/GW 441786 is the main reason. We are not sure whether i.p. injection of GSK-J4/GW 441786 [35] could achieve effective concentrations within the brain tissue in the presence of the BBB whereas intraventricular administration is less suitable when multiple doses are required.

Second, peripheral immune cells play a role in neuroinflammation after SAH but were not specifically studied in this study. The peripheral immune cells like T cells and neutrophil infiltrated from the 24 h after hemorrhage and peaked after 2–7 days [36], so we almost ignored the effect of DHEA on peripheral immune cells in early neuroinflammation (24 h). However, this cannot be ignored in long-term studies.

Third, DHEA content varies with sex and age; therefore, more experiments are needed to explore the effect of DHEA in different situations. Overall, the outstanding safety and tolerability profile of DHEA provides the possibility of rapid clinical application in SAH. We are looking forward to more animal and clinical research of DHEA on microglial activation in SAH.

## Conclusion

Our study found an anti-inflammatory effect of a widely used supplement, DHEA. In vitro experiments indicated an involvement of JMJD3 in this effect. By interacting with TrkA, DHEA induced JMJD3 expression and modulated microglial activation. However, a comprehensive understanding of DHEA in vivo and its therapeutic implications still requires further investigation.

## Supplementary information

**Additional file 1: Table S1.** Primers used in the qPCR. **Table S2.** Antibodies used in the IF and WB. **Figure S1.** Experimental design of this study. Experiment 1, a total of 91 mice were divided into 3 groups in the in vivo experiment: 18 mice in the sham group, 38 mice in the SAH group, and 36 mice in the DHEA-treated SAH group. The mortality rates were 0 (0/18, 0) in sham, 18.42% (7/38) in SAH and 11.11% (4/36) in SAH + DHEA group. 7 and 9 mice were excluded from SAH and SAH + DHEA group respectively. Experiment 2, cultured primary microglia were treated with DHEA or GSK-J4 respectively. Experiment 3, cultured primary microglia were treated with DHEA or GW 441756 respectively. **Figure S2.** Experimental SAH model in C57BL/6 mouse. **Figure S3.** Iba-1 and JMJD3 co-stained microglia cell count after SAH (*n* = 6 fields, one-way ANOVA, **P < 0.01, ***P < 0.001). **Figure S4.** *Jmjd3* gene expression tended to be increased but not significantly in vivo. (one-way ANOVA, *n* = 4, ns, not significant). **Figure S5.** *Jmjd3* responded to haemoglobin (Hb) stimulation in microglia. Primary microglia and neurons were treated with Hb or vehicle medium for 24 h and mRNA expression was detected by qPCR. Ubiquitously transcribed tetratricopeptide repeat, X chromosome (UTX) is a homolog of Jmjd3 and both can be blocked by GSK-J4. **a** In microglia, it is *Jmjd3*, not *UTX*, significantly upregulated after Hb exposure. (one-way ANOVA, *n* = 3, ns, not significant) **b** In neurons, neither expression of *Jmjd3* nor *UTX* changed significantly after Hb exposure. (one-way ANOVA, *n* = 3, ns, not significant, ***P < 0.001)

## Data Availability

The datasets supporting the conclusions of this article are included within the article (and its additional file).

## Abbreviations

BBB: Blood-brain barrier
BDNF: Brain-derived neurotrophic factor
CCK8: Cell viability assay by Cell Counting Kit-8
CCL: C-C motif chemokine ligand
CM: Conditioned medium
CNS: Central nervous system
DAPI: 4,6-Diamidino-2-phenylindole
DHEA: Dehydroepiandrosterone
DHEAS: Dehydroepiandrosterone sulfate
DIV: Day in vitro
DMEM: Dulbecco’s Modified Eagle Medium
EBI: Early brain injury
ELISA: Enzyme-linked immunosorbent assay
FBS: Foetal bovine serum
Hb: Haemoglobin
IF: Immunofluorescence
IL: Interleukin
iNOS: Inducible nitric oxide synthase
Irf: Interferon regulatory factor
JMJD3: Jumonji d3
LPS: Lipopolysaccharide
NLRP: NLR family, pyrin domain-containing
NMDA: N-methyl-D-aspartate
PD: Parkinson’s disease
POD: Postoperative day
qPCR: Real-time PCR
SAH: Subarachnoid haemorrhage
SEM: Standard error of the mean
SPF: Specific pathogen-free
Tgf-β1: Transforming growth factor beta 1
TNF: Tumour necrosis factor
TrkA: Tropomyosin-related kinase A
TUNEL: Terminal deoxynucleotidyl transferase-mediated dUTP nick end labelling
UTX: Ubiquitously transcribed tetratricopeptide repeat, X chromosome
WB: Western blotting
