# Supplementary material for: DHEA Attenuates Microglial Activation via Induction of JMJD3 in Experimental Subarachnoid Haemorrhage
Source: J Neuroinflammation. 2019 Nov 28;16:243. doi: 10.1186/s12974-019-1641-y (PMC6883548; doi:10.1186/s12974-019-1641-y)
Supplement: Supplementary file 1 — Additional file 1: Table S1. Primers used in the qPCR. Table S2. Antibodies used in the IF and WB. Figure S1. Experimental design of this study. Experiment 1, a total of 91 mice were divided into 3 groups in the in vivo experiment: 18 mice in the sham group, 38 mice in the SAH group, and 36 mice in the DHEA-treated SAH group. The mortality rates were 0 (0/18, 0) in sham, 18.42% (7/38) in SAH and 11.11% (4/36) in SAH + DHEA group. 7 and 9 mice were excluded from SAH and SAH + DHEA group respectively. Experiment 2, cultured primary microglia were treated with DHEA or GSK-J4 respectively. Experiment 3, cultured primary microglia were treated with DHEA or GW 441756 respectively. Figure S2. Experimental SAH model in C57BL/6 mouse. Figure S3. Iba-1 and JMJD3 co-stained microglia cell count after SAH (n = 6 fields, one-way ANOVA, **P < 0.01, ***P < 0.001). Figure S4. Jmjd3 gene expression tended to be increased but not significantly in vivo. (one-way ANOVA, n = 4, ns, not significant). Figure S5. Jmjd3 responded to haemoglobin (Hb) stimulation in microglia. Primary microglia and neurons were treated with Hb or vehicle medium for 24 h and mRNA expression was detected by qPCR. Ubiquitously transcribed tetratricopeptide repeat, X chromosome (UTX) is a homolog of Jmjd3 and both can be blocked by GSK-J4. a In microglia, it is Jmjd3, not UTX, significantly upregulated after Hb exposure. (one-way ANOVA, n = 3, ns, not significant) b In neurons, neither expression of Jmjd3 nor UTX changed significantly after Hb exposure. (one-way ANOVA, n = 3, ns, not significant, ***P < 0.001) [file 12974_2019_1641_MOESM1_ESM.docx]

ADDITIONAL FILES

DHEA Attenuates Microglial Activation via Induction of JMJD3 in Experimental Subarachnoid Haemorrhage

Tao Tao^1*^, MD; Guang-jie Liu^2*^, MD; Xuan Shi^3*^, MD; Yan Zhou^2^, MD; Yue Lu^1^, MD, PhD; Yong-yue Gao^2^, MD; Xiang-sheng Zhang^4^, MD, PhD; Han Wang^1^, MD; Ling-yun Wu^1^, MD, PhD; Chun-lei Chen^1^, MD; Zong Zhuang^1^, MD, PhD; Wei Li^1†^, MD, PhD, Chun-hua Hang^1†^, MD, PhD.

1. *Department of Neurosurgery, Nanjing Drum Tower Hospital Clinical College of Nanjing Medical University, Nanjing, Jiangsu Province, China.*
2. *Department of Neurosurgery, Nanjing Drum Tower Hospital, The Affiliated Hospital of Nanjing University Medical School, Nanjing, Jiangsu Province, China.*
3. *Department of Neurology, Jinling Hospital Clinical College of Nanjing Medical University, Nanjing, Jiangsu Province, China.*
4. *Department of Neurosurgery, Beijing Friendship Hospital, Capital Medical University.*

*Drs Tao, Liu and Shi contributed equally.

†Corresponding authors: *Wei Li (E-mail: lwxzlw@126.com; Tel: +86-025-83106666-11903) and Chun-hua Hang (E-mail: hang_neurosurgery@163.com; Tel: +86-025-83106666-11903)*Supplemental Table 1

**Primers used in the qPCR.**

| Gene | Primer Sequence |  |
| --- | --- | --- |
| *Jmjd3* | Forward | TGAAGAACGTCAAGTCCATTGTG |
|  | Reverse | TCCCGCTGTACCTGACAGT |
| *UTX* | Forward | CGGGCGGACAAAAGAAGAAC |
|  | Reverse | CATAGACTTGCATCAGATCCTCC |
| *IL-1β* | Forward | AAGCCTCGTGCTGTCGGACC |
|  | Reverse | TGAGGCCCAAGGCCACAGG |
| *IL-6* | Forward | GAGGATACCACTCCCAACAGACC |
|  | Reverse | AAGTGCATCATCGTTGTTCATACA |
| *TNF-α* | Forward | CAAGGGACAAGGCTGCCCCG |
|  | Reverse | GCAGGGGCTCTTGACGGCAG |
| *NLRP3* | Forward | ATTACCCGCCCGAGAAAGG |
|  | Reverse | CATGAGTGTGGCTAGATCCAAG |
| *IL12* | Forward | AGGTCACACTGGACCAAAGG |
|  | Reverse | TGGTTTGATGATGTCCCTGA |
| *CCL2* | Forward | TGCTGACCCCAATAAGGAA |
|  | Reverse | GCTTGAGGTGGTTGTGGAAAA |
| *iNOS* | Forward | CAGCTGGGCTGTACAAACCTT |
|  | Reverse | CATTGGAAGTGAAGCGTTTCG |
| *Trem2* | Forward | CTGGAACCGTCACCATCACTC |
|  | Reverse | CGAAACTCGATGACTCCTCGG |
| *P2RY12* | Forward | CCCTGTGCGTCAGAGACTAC |
|  | Reverse | CAAGCTGTTCGTGATGAGCC |
| *Arg1* | Forward | CTCCAAGCCAAAGTCCTTAGAG |
|  | Reverse | GGAGCTGTCATTAGGGACATCA |
| *CD206* | Forward | CTCTGTTCAGCTATTGGACGC |
|  | Reverse | CGGAATTTCTGGGATTCAGCTTC |
| *Irf4* | Forward | TCCGACAGTGGTTGATCGAC |
|  | Reverse | CCTCACGATTGTAGTCCTGCTT |
| *Irf7* | Forward | GAGACTGGCTATTGGGGGAG |
|  | Reverse | GACCGAAATGCTTCCAGGG |
| *18 S* | Forward | AGCTTCCGGGAAACCAAAGT |
|  | Reverse | TGTCAATCCTGTCCGTGTCC |

Supplemental Table 2

| **Antibodies used in Immunofluorescence staining** | | | |
| --- | --- | --- | --- |
| anti-Iba-1 | ab5076 | Abcam | Cambridge |
| anti-Jumonji d3 | ab38113 | Abcam | Cambridge |
| anti-CD86 | ab119857 | Abcam | Cambridge |
| anti-CD206 | ab64693 | Abcam | Cambridge |
| anti-NeuN | 26975-1-AP | Proteintech | Wuhan |
| anti-goat Alex Fluor 488-conjugated secondary antibody | ab150129 | Abcam | Cambridge |
| anti-rabbit Alex Fluor 488-conjugated secondary antibody | A11008 | Invitrogen | USA |
| anti-rabbit Alexa Fluor Plus 594-conjugated secondary antibody | A32754 | Invitrogen | USA |
| anti-rat Alexa Fluor 594-conjugated secondary antibody | A21209 | Invitrogen | USA |
| anti-rabbit Alexa Fluor Plus 647-conjugated secondary antibody | A32733 | Invitrogen | USA |
|  | | | |
| **Antibodies used in Western blot** | | | |
| anti-Iba-1 | ab5076 | Abcam | Cambridge |
| anti-Jumonji d3 | ab38113 | Abcam | Cambridge |
| anti-H3K27me3 | ab192985 | Abcam | Cambridge |
| anti-CD86 | ab119857 | Abcam | Cambridge |
| anti-CD206 | ab64693 | Abcam | Cambridge |
| anti-Arg1 | ab91279 | Abcam | Cambridge |
| anti-iNOS | ab15323 | Abcam | Cambridge |
| anti-phospho-TrkA (tyr490) | 9141 | CST | Danvers |
| anti-TrkA | ab76291 | Abcam | Cambridge |
| anti-Akt | 4691 | CST | Danvers |
| anti-phospho-Akt | 9271 | CST | Danvers |
| and anti-β-actin | BS6007M | Bioworld | Nanjing |


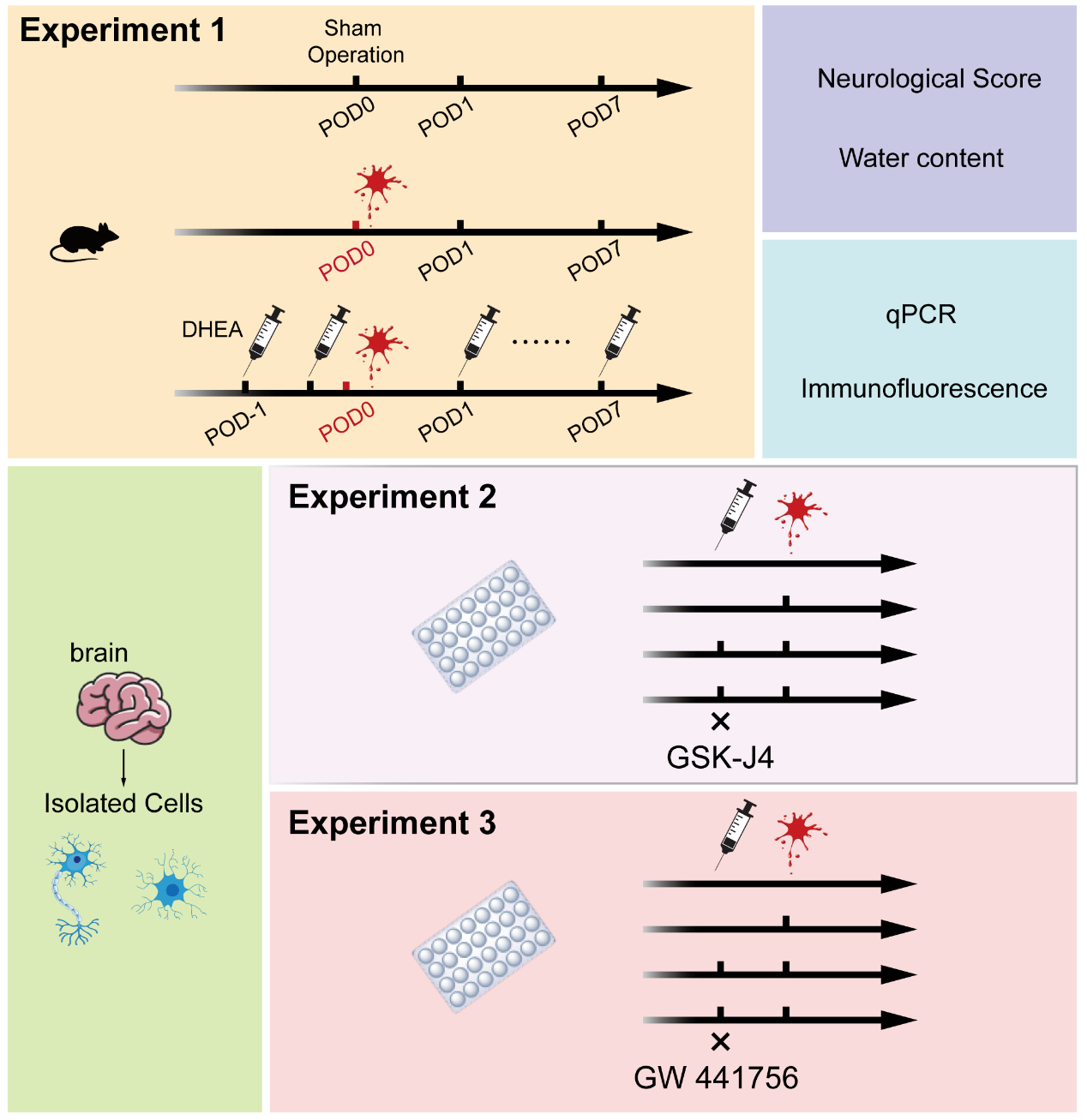


**Supplemental Figure 1:** Experimental design of this study.

Experiment 1, a total of 91 mice were divided into 3 groups in the *in vivo* experiment: 18 mice in the sham group, 38 mice in the SAH group, and 36 mice in the DHEA-treated SAH group. The mortality rates were 0 (0/18, 0) in sham, 18.42% (7/38) in SAH and 11.11% (4/36) in SAH+DHEA group. 7 and 9 mice were excluded from SAH and SAH+DHEA group respectively. Experiment 2, cultured primary microglia were treated with DHEA or GSK-J4 respectively. Experiment 3, cultured primary microglia were treated with DHEA or GW 441756 respectively.


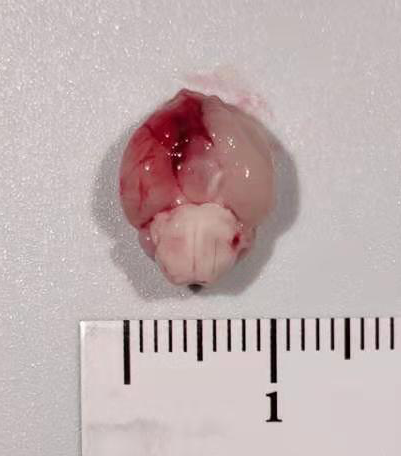


Supplemental Figure 3: Experimental SAH model in C57BL/6 mouse.

**Supplemental Fig. 3,** Iba-1 and JMJD3 co-stained microglia cell count after SAH (n=6 fields, one-way ANOVA, ***P<*0.01, ****P*<0.001).

Supplemental Figure 4: *Jmjd3* gene expression tended to be increased but not significantly *in vivo*. (one-way ANOVA, n=4, ns, not significant)

**Supplemental Figure 5:** *Jmjd3* responded to haemoglobin (Hb) stimulation in microglia.

Primary microglia and neurons were treated with Hb or vehicle medium for 24 hours and mRNA expression was detected by qPCR. Ubiquitously transcribed tetratricopeptide repeat, X chromosome (UTX) is a homolog of Jmjd3 and both can be blocked by GSK-J4. **A**, In microglia, it’s *Jmjd3*, not *UTX*, significantly upregulated after Hb exposure. (one-way ANOVA, n=3, ns, not significant) **B**, In neurons, neither expression of *Jmjd3* nor *UTX* changed significantly after Hb exposure. (one-way ANOVA, n=3, ns, not significant, ****P*<0.001)

Supplemental Reference

1. Lu Y, Zhang XS, Zhou XM, Gao YY, Chen CL, Liu JP, et al. Peroxiredoxin 1/2 protects brain against h2o2-induced apoptosis after subarachnoid hemorrhage. *Faseb j*. 2018:fj201801150R

2. Lu Y, Zhang XS, Zhang ZH, Zhou XM, Gao YY, Liu GJ, et al. Peroxiredoxin 2 activates microglia by interacting with toll-like receptor 4 after subarachnoid hemorrhage. *J Neuroinflammation*. 2018;15:87
